# Correction: Castelan-Ramírez et al. Schwann Cell Autophagy and Necrosis as Mechanisms of Cell Death by *Acanthamoeba*. *Pathogens* 2020, *9*, 458

**DOI:** 10.3390/pathogens13100852

**Published:** 2024-09-30

**Authors:** Ismael Castelan-Ramírez, Lizbeth Salazar-Villatoro, Bibiana Chávez-Munguía, Citlaltepetl Salinas-Lara, Carlos Sánchez-Garibay, Catalina Flores-Maldonado, Dolores Hernández-Martínez, Verónica Anaya-Martínez, María Rosa Ávila-Costa, Adolfo René Méndez-Cruz, Maritza Omaña-Molina

**Affiliations:** 1 Posgrado en Ciencias Biológicas, Universidad Nacional Autónoma de Mexico (UNAM), Av. Ciudad Universitaria 3000, Coyoacán P.C. 04510, Mexico; ismaelc.40@gmail.com; 2 Laboratorio de Amibas Anfizoicas, Facultad de Estudios Superiores Iztacala (FESI), Medicina, UNAM, Tlalnepantla 54090, Mexico; alol_madole@yahoo.com.mx; 3 Departamento de Infectómica y Patogénesis Molecular, CINVESTAV-IPN, Ciudad de Mexico 07360, Mexico; bioagam@hotmail.com (L.S.-V.); bchavez@cinvestav.mx (B.C.-M.); 4 Departamento de Neuropatología, Instituto Nacional de Neurología y Neurocirugía “Manuel Velasco Suárez”, Ciudad de Mexico 14269, Mexico; cisala69@hotmail.com (C.S.-L.); carlos.s.garibay@live.com.mx (C.S.-G.); 5 Laboratorio de Histología y Patología, FESI, Medicina, UNAM, Tlalnepantla 54090, Mexico; 6 Departamento de Fisiología, Biofísica y Neurociencias, CINVESTAV–IPN, Ciudad de Mexico 07360, Mexico; ceflores@fisio.cinvestav.mx; 7 Centro de Investigación en Ciencias de la Salud, Facultad de Ciencias de la Salud, Universidad Anáhuac, Huixquilucan C.P. 52786, Mexico; anayamtz@yahoo.com.mx; 8 Departamento de Neurociencia, FESI, UNAM, Tlalnepantla 54090, Mexico; nigraizo@unam.mx; 9 Laboratorio de Inmunología, FESI, UNAM, Tlalnepantla 54090, Mexico; renemen@gmail.com

## Error in Figure 5

In the original publication [1], Figure 5c showed an image that was edited to eliminate an electron-dense artifact (precipitate stain generated using the contrast technique in electron microscopy), which in no way modified the essence of what the authors wanted to highlight in the publication. However, the authors now consider it more appropriate to present the image highlighting only the area of interest.

The corrected Figure 5c appears as follows:

**Figure 5:**
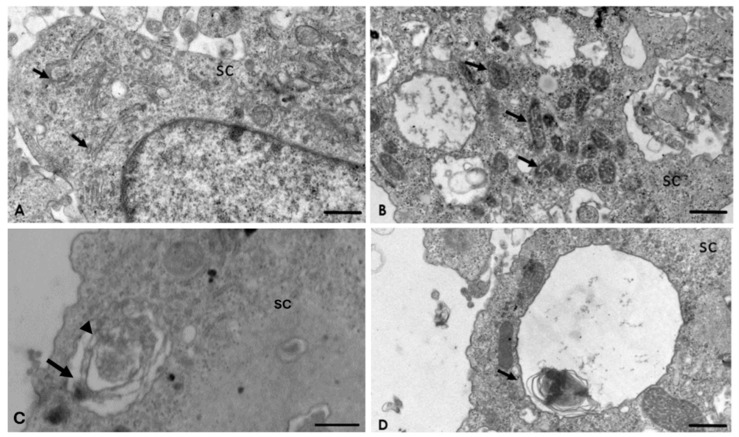
Interaction of *A. culbertsoni* and Schwann cells (SC) after 2 h of interaction. (**A**,**B**) Ultrastructural alterations in organelles of SC were frequently observed (arrows) in the rough endoplasmic reticulum (**A**) and mitochondria (**B**) Bars = 2 µm. (**C**,**D**) Multilamellar bodies with cytoplasmic material (arrows) persisted. In electron micrograph (**C**) Bar = 500 nm, a double membrane (arrow head) is evident, characteristic of these structures. Bar = 2 µm.

## Addition of Supplementary File

The authors would like to include the following supporting information in the main text, under Section 2.4, “Analysis of the Interaction of *A. culbertsoni* Trophozoites with SC through TEM,” in the third paragraph. 

The reference to the supplementary materials should be added to the third paragraph of Section 2.4. The sentence, “Multilamellar bodies with the characteristic double membrane persisted (Figure 5C),” should be revised to, “Multilamellar bodies with the characteristic double membrane persisted (Figure 5C and Supplementary Figure S1).”

In the Supplementary Materials section at the end of the paper, the authors included the following:

**Supplementary Materials:** The following supporting information can be downloaded at: http://www.mdpi.com/2076-0817/9/6/458/s1, Figure S1: Original image corresponding to Figure 5C in which Multilamellar body with cytoplasmic material (arrow) persisted are described.

The Figure S1 appears as follows:

**Figure S1:**
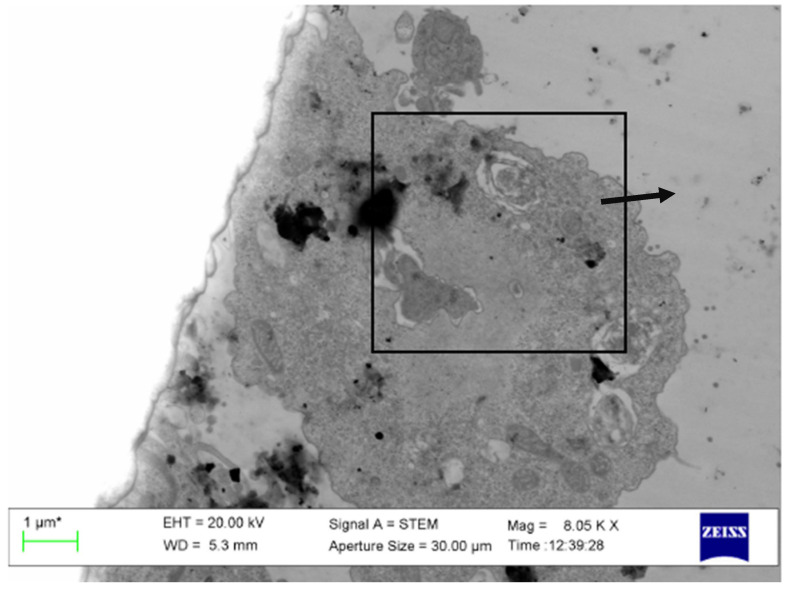
Original image corresponding to Figure 5C in which Multilamellar body with cytoplasmic material (arrow) persisted are described.

The authors state that the scientific conclusions are unaffected. These corrections were approved by the Academic Editor. The original publication has also been updated.
