# Supplementary material for: Schwann Cell Autophagy and Necrosis as Mechanisms of Cell Death by Acanthamoeba
Source: Pathogens. 2020 Jun 9;9(6):458. doi: 10.3390/pathogens9060458 (PMC7350333; doi:10.3390/pathogens9060458)
Supplement: Supplementary file 1 [file pathogens-09-00458-s001.zip › pathogens-804459-Supplementary.pdf]

Supplementary Materials

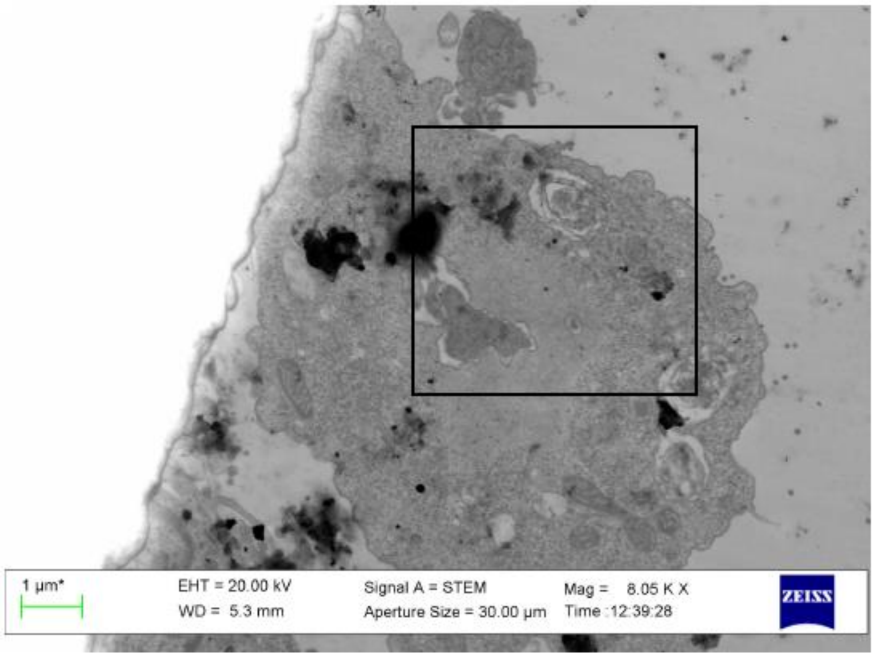

Figure S1. Original image corresponding to figure 5C in which Multilamellar body with cytoplasmic material (arrow) persisted are described.
